# Relationship between the Branching Patterns of the Radial Nerve and Supinator Muscle

**DOI:** 10.1155/2021/8691114

**Published:** 2021-10-14

**Authors:** Anna Jeon, Ye-Gyung Kim, Seong-Oh Kwon, Je-Hun Lee

**Affiliations:** ^1^Department of Anatomy, College of Medicine, Ewha Womans University, Seoul, Republic of Korea; ^2^Department of Anatomy, College of Medicine, Chung-Ang University, Seoul, Republic of Korea; ^3^Department of Neurosurgery, CHA Gumi Medical Center, CHA University, Pocheon, Republic of Korea; ^4^Korea Institute for Applied Anatomy, College of Sports Science, Korea National Sport University, Seoul, Republic of Korea

## Abstract

The posterior interosseous nerve (PIN) innervates the posterior compartment muscle of the forearm and is a continuation of the deep branch of the radial nerve. The anatomic descriptions of PIN vary among different authors. This study investigated the distribution patterns of PIN and its relationships to the supinator muscle. This study investigated which nerves innervate the posterior compartment muscles of the forearm, the radial nerve, and the PIN, using 28 nonembalmed limbs. Also, the points where the muscle attaches to the bone were investigated. The measured variables in this study were measured from the most prominent point of the lateral epicondyle of the humerus (LEH) to the most distal point of the radius styloid process. For each specimen, the distance between the above two points was assumed to be 100%. The measurement variables were the attachment area of the supinator and branching points from the radial nerve. The attachment points of the supinator to the radius and ulna were 47.9% ± 3.6% and 31.5% ± 5.2%, respectively, from the LEH. In 67.9% of the specimens, the brachioradialis and extensor carpi radialis longus (ECRL) were innervated by the radial nerve before superficial nerve branching, and the extensor carpi radialis brevis (ECRB) innervated the deep branch of the radial nerve. In 21.4% of the limbs, the nerve innervating the ECRB branched at the same point as the superficial branch of the radial nerve, whereas it branched from the radial nerve in 7.1% of the limbs. In 3.6% of the limbs, the deep branch of the radial nerve branched to innervate the ECRL. PIN was identified as a large branch without divisions in 10.7% and as a deep branch innervating the extensor digitorum in 14.3% of the limbs. The anatomic findings of this study would aid in the diagnosis of PIN syndromes.

## 1. Introduction

The posterior interosseous nerve (PIN) innervates the posterior compartment muscles of the forearm and is a continuation of the deep branch of the radial nerve. PIN is termed so for its emergence from between the 2 heads of the supinator muscle. Before the radial nerve passes through the supinator, it is commonly identified as the deep branch of the radial nerve [1, 2]. The muscles in the posterior compartment of the forearm are innervated by the radial nerve; anatomy textbooks provide more detailed information on the origin of specific nerves innervating those muscles.

However, some anatomy textbooks provide slightly differing descriptions of PIN. *Gray's Anatomy* [1] describes the brachioradialis (BR) and extensor carpi radialis longus (ECRL) as being innervated by the radial nerve before branching into the superficial and deep branches. The extensor carpi radialis brevis (ECRB) was described to be innervated by the deep branch of the radial nerve before penetrating the supinator muscle. The authors explained that PIN innervated the extensor digiti minimi (EDM), extensor digitorum (ED), and extensor carpi ulnaris. Another textbook [2] described BR and ECRL to be innervated by the radial nerve. The ECRB, ED, EDM, supinator, and ECU were described as being innervated the deep branch of the radial nerve. The extensor indicis (EI), abductor pollicis longus (APL), extensor pollicis longus (EPL), and extensor pollicis brevis (EPB) were described to be innervated by PIN. Considering the varying descriptions, anatomic research is required to identify the correct anatomic structure, which may aid in diagnosis and management of nerve entrapment syndrome. Of note, anatomic variability is critical to medical practice and should be considered by clinicians [3].

Anatomic studies on the association between PIN and the supinator muscle exist [4–14]. However, detailed information on the precise point of branching out of the radial nerve to innervate each posterior compartment muscle in the forearm with the superficial branch is needed. Thus, this study is aimed at conducting a detailed anatomic study on the nerve branches emerging from the radial nerve of the forearm.

## 2. Materials and Methods

Fourteen embalmed adult cadavers (males, 7; females, 7; age range 63-95 years) were dissected for this study. All cadavers used in this study were legally donated to medical school. This study was conducted in accordance with the Declaration of Helsinki.

**Figure 1:**
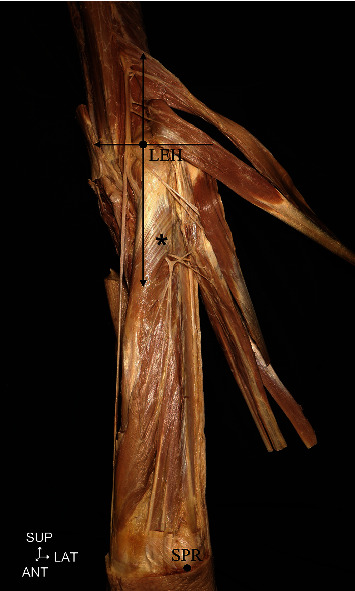
Measurement method with the forearm in the supinated position. LEH: lateral epicondyle of the humerus; SPR: styloid process of the radius; SUP: superior; LAT: lateral; ANT: anterior; asterisk: supinator.

**Figure 2:**
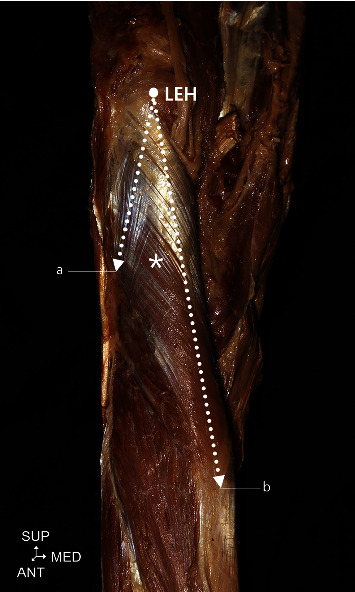
Supinator muscle attachment. (a) Distal attachment of the supinator at the ulna. (b) Distal attachment of the supinator at the radius. LEH: lateral epicondyle of the humerus; asterisk: supinator.

Limbs showing evidence of surgery or injury around the forearm region were excluded. To measure variables associated with the branching of the radial nerve, the most prominent point of the lateral epicondyle of the humerus (LEH) and the most distal point of the styloid process of the radius (SPR) were identified before dissection. A line connecting the LEH and the SPR was used as the reference line. For each specimen, the distance between the above two points was assumed to be 100%. The *y* coordinate was extended and measured in the direction of the arm and marked as the (+) value. Conversely, the same measurement but towards the forearm was marked as the (–) value (Figure 1). Only the radial and ulnar attachment points of the supinator muscle were measured directly from the LEH (Figure 2). The reference point was expressed as the absolute distance along the reference line using the LEH as the starting point.

For the dissection, only the skin was removed to expose the hypodermis, and the superficial fascia was then carefully removed to identify the neurovascular structures on the posterior elbow region. Further careful dissection was performed to identify the nerve branches around the supinator by removing some extensor muscles. After the dissection of the nerve branches around the supinator, the nerve branch points around the muscular and cutaneous branches of the radial nerve were investigated. The measurement variables were as follows:

1. The reference line between the LEH and SPR
2. The attachment points of the supinator to the radius and ulna from the LEH
3. The branching points of each posterior forearm muscle from the LEH, and
4. The transverse distance from the radial nerve to the LEH.

The measurements were conducted with the forearm in the supinated position. A single observer obtained all measurements using a measuring tape and digital callipers (resolution 0.01 mm, CD-20PSX, Mitutoyo, Japan). The data were analysed using SPSS version 23.0 (IBM SPSS Inc., Chicago, IL, USA).

## 3. Results

The mean distance of the reference line from the LEH to the SPR was 247.3 ± 15.7 mm. The distances from the reference line did not differ significantly between males and females or between the right and left sides (*p* ≥ 0.05).

**Figure 3:**
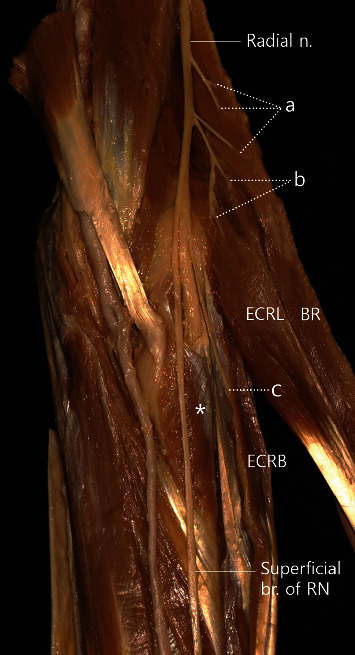
A photograph showing radial nerve branching with muscles. (a) Muscular branches for the brachioradialis (BR). (b) Muscular branches for the extensor carpi radialis longus (ECRL). (c) Extensor carpi radialis brevis (BCRB). Asterisk: supinator; n: nerve; br. of RN: branch of the radial nerve.

The attachment points of the supinator to the radius and ulna were –118.3 ± 10.9 mm (47.9 ± 3.6%) and –77.8 ± 13.6 mm (31.5 ± 5.2%), respectively, from the LEH (Table 1 and Figure 2). In all specimens, the BR and ECRL had (+) *y* coordinate values. The branch points of the radial nerve for BR and ECRL were 20.3 ± 3.2% and 13.9 ± 4.1%, respectively (Table 2 and Figure 3). With regard to ECRB, in 25.0% of the specimens, the nerve branched superiorly to the reference line, with a mean value of 4.0 ± 5.2%. In contrast, in 36.0% of the specimens, the nerve branched inferiorly to the reference line, with an average value of –13.3 ± 7.6%. Furthermore, in 39.0% of the specimens, the nerve branched on the reference line (Table 2 and Figure 3).

**Figure 4:**
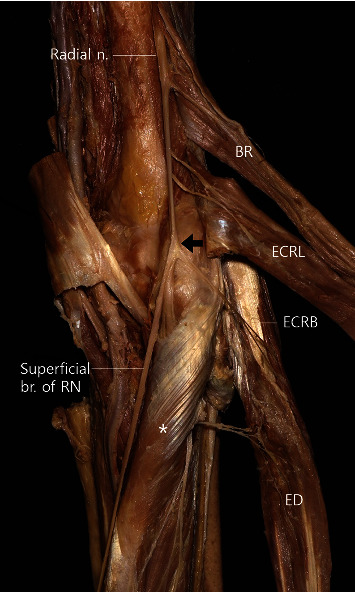
A photograph showing the nerve branch (arrow) for the extensor carpi radialis brevis (ECRB) and superficial branch of the radial nerve (superficial br. of RN). BR: brachioradialis; ECRL: extensor carpi radialis longus; ECRB: extensor carpi radialis brevis; ECU: extensor carpi ulnaris; asterisk: supinator.

**Figure 5:**
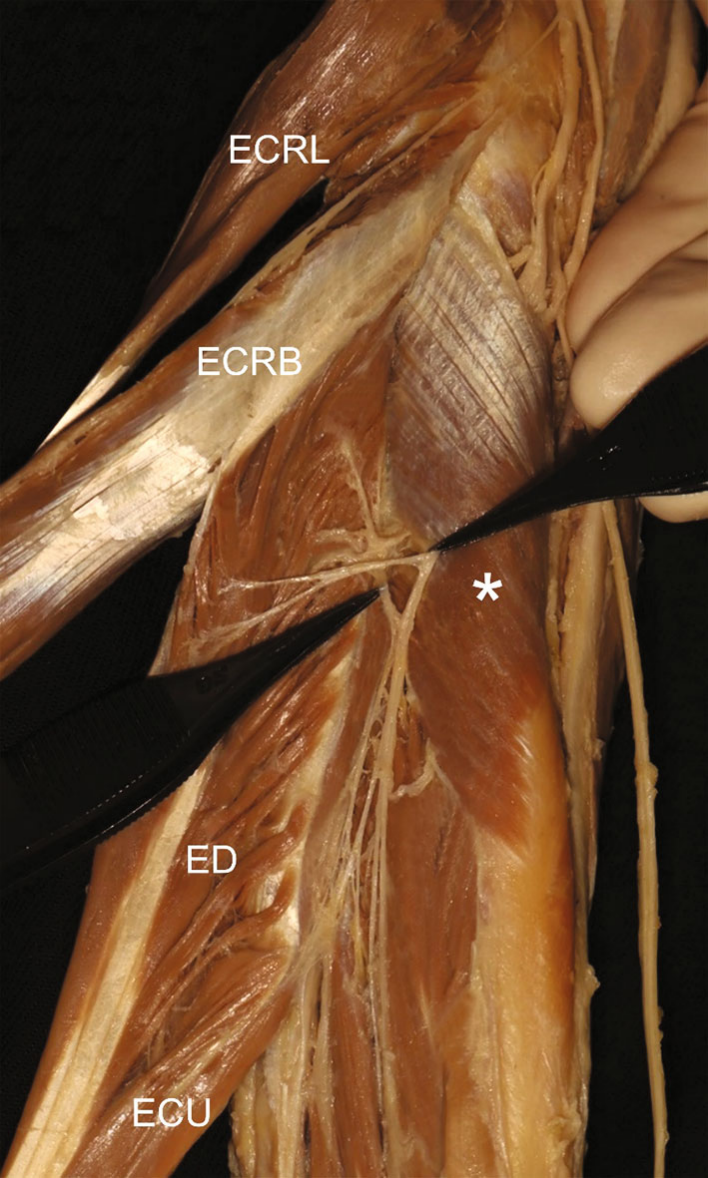
A photograph showing the nerve branch for the extensor carpi radialis brevis (ECRB) and the nerve branch from the deep branch of the posterior interosseous nerve (PIN) to innervate the extensor digitorum (ED). ECRL: extensor carpi radialis longus; ECU: extensor carpi ulnaris; asterisk: supinator.

**Figure 6:**
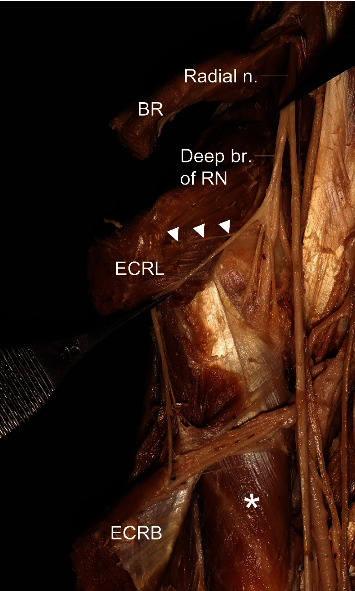
A photograph showing the nerve branch (arrow) for the extensor carpi radialis longus (ECRL). BR: brachioradialis; ECRB: extensor carpi radialis brevis. Deep br. of RN: deep branch of radial nerve; asterisk: supinator.

We identified the branching points of the radial nerve after determining which muscle it innervated. In 67.9% of specimens, the BR and ECRL were innervated by the radial nerve before superficial nerve branching, and the ECRB was innervated by the deep branch of the radial nerve (Figure 3). In 21.4% of all specimens, the nerve innervating the ECRB branched out of the same point as the superficial branch of the radial nerve (Figure 4). In 7.1% of the specimens, the nerve branched out from the radial nerve (Figure 5). In 1 specimen (3.6%), the deep branch of the radial nerve branched to innervate the ECRL (Figure 6).

**Figure 7:**
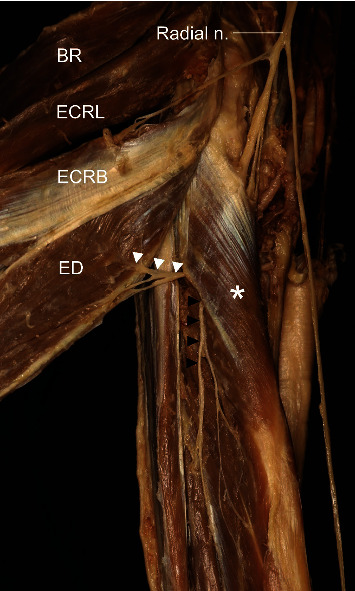
A photograph showing the posterior interosseous nerve branch (PIN). BR: brachioradialis; ECRL: extensor carpi radialis longus; ECRB: extensor carpi radialis brevis; asterisk: supinator; ED: extensor digitorum; white arrow: superficial branch of the PIN; black arrow: deep branch of the PIN; asterisk: supinator.

**Figure 8:**
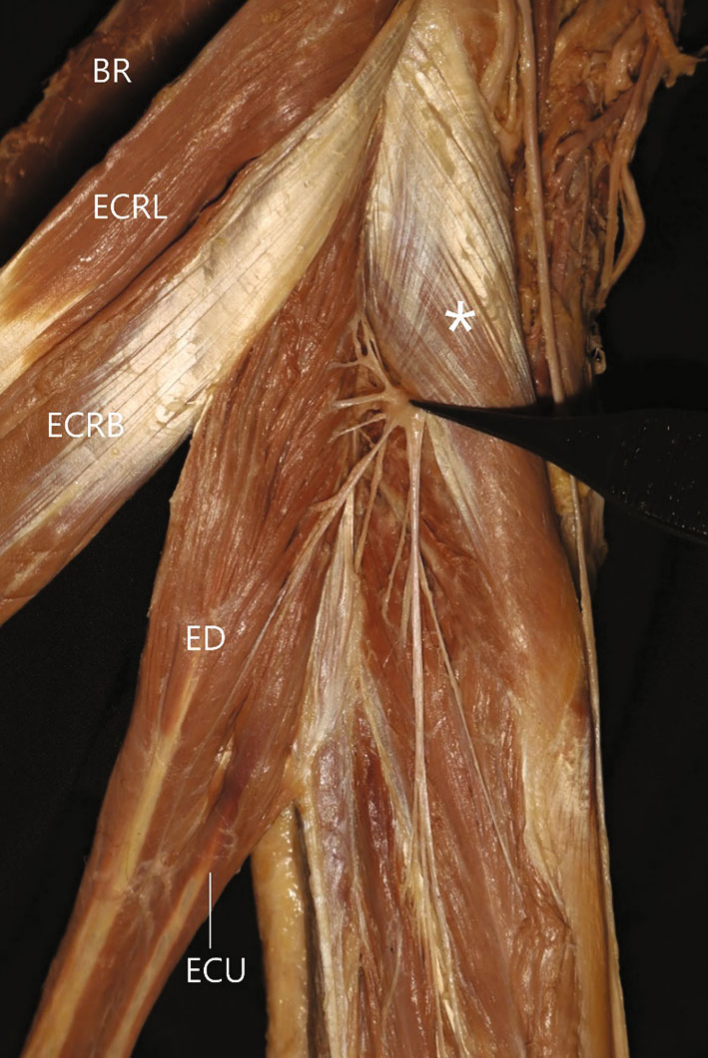
A photograph showing the common trunk of the posterior interosseous nerve. BR: brachioradialis; ECRL: extensor carpi radialis longus; ECRB: extensor carpi radialis brevis; asterisk: supinator; ED: extensor digitorum; ECU: extensor carpi ulnaris; asterisk: supinator.

**Figure 9:**
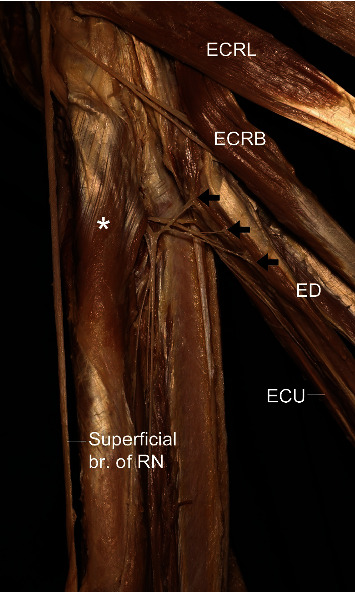
A photograph showing the deep branch (arrow) of the posterior interosseous nerve innervating the extensor digitorum (ED). BR: brachioradialis; ECRL: extensor carpi radialis longus; ECRB: extensor carpi radialis brevis; ECU: extensor carpi ulnaris; superficial br. of RN: superficial branch of the radial nerve; asterisk: supinator.

**Figure 10:**
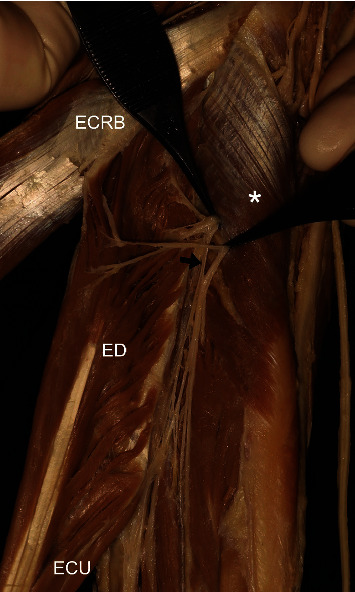
A photograph showing the communicating branch of (arrow) the posterior interosseous nerve. ECRB: extensor carpi radialis brevis; ED: extensor digitorum; ECU: extensor carpi ulnaris; asterisk: supinator.

In most cases (67.9%), after the radial nerve penetrated the supinator, it segregated into 2 large branches (superficial and deep branch) in the supinator and innervated the posterior compartment muscles of the forearm. Thus, the superficial branch innervated the superficial muscles such as the ED and EDM, and the deep branch innervated deep muscles such as the EI, APL, EPB, and EPL (Figure 7). However, some varying nerve branching patterns were also observed. One large branch was found to innervate all muscles without nerve division in 10.7% of the specimens. In 14.3% of the specimens, the deep branch innervated the ED (Figures 8 and 9). We observed some communicating nerve branches between the superficial and deep branches (7.1%) (Figure 10).

## 4. Discussion

A study using cadaver dissection [5] reported that the ECRB was innervated by the radial nerve, superficial radial nerve, and PIN in 45.0%, 29.0%, and 26.0% of the specimens, respectively. Our study focused on identifying the level at which the radial nerve branched to innervate the ECRB. There were many specimens (75.0%) where the nerve branch emerged below the *x* coordinate line. However, on further examination, the nerve originated from the deep branch, the superficial radial nerve, and the radial nerve in 53.3%, 26.7%, and 20.0% of the specimens, respectively. A previous study [5] reported that the nerve innervating the ECRB branched from the superficial radial nerve. However, no branches from the superficial radial nerve were observed in this study. The radial nerves were also named differently in this study compared to the previous studies.

The distribution of branches at a certain point on the radial nerve is important information for surgery. A previous study [5] reported a distance of 1.0 ± 0.3 cm from the biceps tendon; however, in our study, the distance was measured from the LEH in the supinated position, and the average distance was 2.2 ± 0.3 cm. These results were helpful in clinical applications such as distal biceps repair drilling [4] or nerve transfer surgery [15]. Another anatomic study that attempted to identify a safe zone for arthroscopic surgery of the anterior elbow showed that forearm pronation widens the safe zone [16]. A study assessed the position of the radial nerve using a posterior approach [17]. This study also assessed the association between the position of the radial nerve and the surrounding structures. Although we did not study the change in the position of the radial nerve according to pronation or supination of the forearm, changes in nerves according to the movement or the location of nerves through various approaches need to be studied.

A study using medical images [18] reported that the PIN syndrome is often caused by the radial nerve trunk at the upper arm level rather than the supinator. Furthermore, a study highlighted the importance of neuroimaging methods for better diagnosis of fascicular torsion of the PIN [19]. The results of this study, along with those of the previous studies, provide the anatomic data needed for accurate diagnosis. In this study, we segregated the radial nerve into subdivisions basis the location of the nerve branch. The radial nerve continued to be identified till the emergence of the superficial cutaneous branch; it was identified as the deep branch of the radial nerve between the branching point of the superficial cutaneous nerve and the entry point into the supinator. Finally, the radial nerve was identified as PIN after passing the supinator. In entrapment syndrome, the supinator muscle is often not the cause. Our results should contribute to the knowledge on palpation of the supinator for diagnosis. Our study identified each nerve branching point from the radial nerve according to the reference line. These metrical results can aid in identifying the location of nerve entrapment and provide basic information for the location of injury even when diagnosing using ultrasound.

Two previous studies also discussed nerve entrapment syndrome around the supinator [20, 21]. Naik et al. [21] reported that the radial nerve and PIN were often compressed around the supinator; per this study, PIN syndrome and radial tunnel syndrome have similar implications, and both result from nerve compression at the proximal elbow. Ceri et al. [20] investigated the arcade of Frohse around the supinator. All the sites discussed among several studies are related to the supinator. In this study, BR and ECRL were innervated by nerves branching from the radial nerve, and the ECRB was innervated by nerves branching from the deep branch of the radial nerve in most specimens. The results of this study indicate that the branching point of BR is 7% proximal than ECRL on an average. With regard to ECRB, many specimens were innervated by the radial nerve at the same point of the superficial cutaneous nerve, and the nerve branched out from the deep branch of the radial nerve (Table 2 and Figure 3). In most cases (67.9%), immediately after PIN passed through the supinator or within that muscle, it split into 2 main branches to innervate the superficial and deep compartment muscles of the posterior compartment of the forearm (Figure 7). We observed that the ECRB branched out superiorly to the reference line in 25% of the specimens; we believe these results will aid in muscle evaluation in supinator-related syndromes. Finally, our results indicate that treating supinator-related syndromes 48.0% and 32.0% from the LEH towards the radius and the ulna, respectively, using conservative treatment methods may help in better treatment (Figure 2).

## Figures and Tables

**Table 1:** Attachment region of the supinator.

Side	Mean ± SD (mm)	Mean ± SD (%)
Radius	–118.3 ± 10.9	47.9 ± 3.6
Ulna	–77.8 ± 13.6	31.5 ± 5.2

**Table 2:** Nerve branching point from the radial nerve to muscle innervation.

Muscles		Mean ± SD (mm)	Mean ± SD (%)
BR		49.9 ± 6.9	20.3 ± 3.2
ECRL		33.8 ± 9.0	13.9 ± 4.1
ECRB	(superiorly to the RL)	10.9 ± 14.5	4.1 ± 5.2
	(inferiorly to the RL)	–13.3 ± 7.6	–5.3 ± 3.1

RL: reference line.

## Data Availability

The data of this study used to support the finding of this study are included within the article.
